# Molecular Toxicology of Substances Released from Resin–Based Dental Restorative Materials

**DOI:** 10.3390/ijms10093861

**Published:** 2009-09-04

**Authors:** Athina Bakopoulou, Triantafillos Papadopoulos, Pavlos Garefis

**Affiliations:** 1 Department of Fixed & Implant Prosthodontics, School of Dentistry, Aristotle University of Thessaloniki, Thessaloniki 541 24, Greece; E-Mails:athinabakopoulou@hotmail.com (B.A.);garefis@dent.auth.gr (G.P.); 2 Department of Biomaterials, School of Dentistry, National and Kapodistrian University of Athens, Athens, 115 27, Greece

**Keywords:** dental composite resins, molecular toxicology, biocompatibility, biodegradation, cytotoxicity, genotoxicity

## Abstract

Resin-based dental restorative materials are extensively used today in dentistry. However, significant concerns still remain regarding their biocompatibility. For this reason, significant scientific effort has been focused on the determination of the molecular toxicology of substances released by these biomaterials, using several tools for risk assessment, including exposure assessment, hazard identification and dose-response analysis. These studies have shown that substances released by these materials can cause significant cytotoxic and genotoxic effects, leading to irreversible disturbance of basic cellular functions. The aim of this article is to review current knowledge related to dental composites’ molecular toxicology and to give implications for possible improvements concerning their biocompatibility.

## 1. Introduction

The development and widespread use of new generations of resin-based dental restorative materials has allowed for the application of more conservative, esthetic and long lasting restorative techniques. These adhesive techniques are extensively used in a wide variety of applications in dentistry, including restorative procedures, prosthodontics, orthodontics and preventive dentistry, making resin-based composites one of the most important groups of materials in dental practice. The main bulk of scientific and manufacturing effort during the past years has been focused on the improvement of the filler fraction of these materials, providing a great variety of new formulations in the micro- or nano-scale, in an attempt to improve their mechanical and esthetic properties. On the other hand, little improvement has been offered with respect to the resinous matrix of these materials, which is based in the majority of commercially available products on methacrylate monomers. Most of these products consist of a mixture of various methacrylate monomers, such as BisGMA (2,2-bis[4-(2-hydroxy-3-methacryloxypropoxy)phenyl]propane) and UDMA (urethane dimethacrylate) in combination with comonomers of lower viscosity, such as TEGDMA (triethyleneglycol dimethacrylate), EGDMA (ethyleneglycol dimethacrylate) or DEGDMA (diethyleneglycol dimethacrylate) [1–3]. These methacrylate monomers, polymerized through radical chain polymerisation, are responsible for major clinical disadvantages, such as polymerization shrinkage of the composites, leading to microleakage phenomena in the tooth-material interface [4,5], as well as adverse effects caused by substances released from the resinous matrix due to incomplete polymerization or resin degradation [6–9]. Several attempts have been made in order to overcome these problems through the development of new monomer systems, including the so called “expanding monomers”, based on spiro-orthocarbonate molecules [10], epoxides systems (oxiranes, siloranes) set via cationic polymerization [11,12], or multifunctional hyper-branched methacrylic monomers (dendrimers), as alternatives to the conventional methacrylic formulations [13–15]. However, the insufficient mechanical properties of these systems, together with several problems concerning their filler incorporation and polymerization have not yet allowed for their extensive clinical application.

The release of methacrylic monomers together with compounds of the polymerization system from dental composites has been considered as a source of a wide variety of adverse biological reactions, including local and systemic toxicity, pulp reactions, allergic and estrogenic effects. These effects have been extensively reviewed in the literature [16–21]. On the other hand, a significant amount of scientific effort has been focused during the past few years by several research groups in the world on the determination of the molecular mechanisms underlying the dental composites’ toxicological effects (cytotoxicity and genotoxicity). These studies have used a variety of sophisticated molecular biological techniques in order to assess the potential risks that these chemicals could pose to the living tissues, including exposure assessment, hazard identification, dose-response analysis, analysis of signaling pathways implicated in tissue response and repair and genotoxicity analysis, as a tool for potential mutagenic and clastogenic effects. This rapidly growing field of molecular toxicology of substances released by dental restorative materials also reflects the expanding public awareness of potential health risks caused by these materials during their long term clinical services and the urgent need for improvement on their biological properties. Therefore, the aim of this article is to review the rapidly growing body of knowledge related to dental composite materials’ molecular toxicology and to give implications for possible future improvements with respect to their biocompatibility.

## 2. Nature, Amount and Bioavailability of Substances Released by Resin–Based Dental Restorative Materials

Dental composite resin materials contain polymer networks that have been shown to be susceptible to hygroscopic and hydrolytic effects to varying extents, dependent upon their chemistry and structure. These effects may not only affect their physical and mechanical properties leading to a shortened service life but they may also be responsible for short-term release of unreacted components, as well as long-term elution of degradation products in the oral cavity [8,16,22,23].

The elution of unreacted components from dental composites is influenced by several factors, including the chemistry of the composite (mainly the solubility and the molecular weight of the monomers used), the degree of conversion, the degree of crosslinking of the polymer network, the surface treatment of the filler particles and the nature of the solvent [7,23–26]. The free radical polymerization of dimethacrylate monomers produces a highly crosslinked polymer network, but also leaves unreacted monomers or oligomers. For most resin-based composites the degree of conversion has been reported to vary from 55 to 75 % when they are directly cured by halogen or LED curing units [26–32] and can reach up to 80% when the composite resins are further post-cured indirectly under different laboratory conditions, including high intensity light, heat, pressure or a combination of the above [33–35]. On the other hand, the degree of conversion can be as low as 25–35% if oxygen is in contact with the resin surface during the setting reaction (oxygen inhibition layer), allowing for more unreacted components to being released from the polymer network [24,36]. The latter may constitute a significant biological risk.

The nature and amount of released components has been evaluated by several elution studies, using a variety of techniques, including Ultraviolet (UV) and Infrared (IR) Radiation, High Performance Liquid Chromatography (HPLC) and Gas Chromatography/Mass Spectroscopy (GC/MS) [6,8,36–52]. These studies have shown that resin-based dental materials are able to release more than 30 different compounds into aqueous or organic solvents. These substances include major (co)-monomers, additives, compounds of the polymerization system (co initiators, stabilizers, or inhibitors), as well as ions form the filler particles. Most of the above mentioned studies support that almost any component present in a composite or adhesive resin is capable of being leached from the set material [42,49]. Generally, extraction is more complete in alcohol or organic solvents as compared to water. Moreover, solution of 75% ethyl alcohol in water, which is recommended by US FDA as a food/oral simulating liquid, has been shown to be among the best solvents for dental composite networks, although this solution extracts far more organic compounds, as compared to the artificial saliva that is composed of a complex salt mixture [6,53,54]. However, there are a few components that are also leached into an aqueous media. In particular, considerable amounts of TEGDMA and HEMA may be released by polymerized composite resins into water. Bis-GMA, UDMA, TEGDMA, EGDMA DEGDMA, 1,6-hexanediol di-methacrylate, methyl methacrylate, camphoroquinone, 4-*N,N*-dimethylaminobenzoic acid, ethyl ester, and various other substances have been also identified in minor concentrations in aqueous extracts [16,42,44,45,50,54]. In filled polymers, ions from the filler particles may also be released. These include strontium, silicon, boron, sodium and barium, depending on the filler type [55,56].

It is important to note that not all unreacted methacrylate groups in resin-based materials are capable of being leached into aqueous environment, because they are part of dimethacrylate molecules covalently bound to one end of the main polymer chain. It has been reported that approximately 10% or less of the nonreacted methacrylate groups exist as residual monomer and are available to be leached into various media [6,57]. Studies have shown that elution of as little as 0.05% to as high as 2.0 % of the weight of the specimen into aqueous media, with elution into alcohol and other organic solvents being generally higher (2–6%) [6,8,41,46,57]. In most cases, the elution process is completed within the first few days or weeks after initial polymerization depending on the solvent [23,53,58]. Among the resinous monomers released, hydrophilic monomers, such as TEGDMA, were identified in higher amounts into aqueous extraction media (0.04–2.3%wt) as compared to BisGMA (0.03–0.07%) [19,24,42,59,60]. Moreover, the hydrophilic monomers HEMA and TEGDMA were the only ones to be able to diffuse through the dentin into the pulp space at significantly high concentrations in the millimolar range. The diffusion increases when the remaining dentin thickness is decreased, especially below 1mm or after acid etched treatment [61]. HEMA leaching from dental adhesives might reach concentrations as high as 1.5–8 mmol/L in the pulp [62], whereas TEGDMA concentrations could be in the range of 4 mmol/L [20,63]. These concentrations may be high enough to cause detrimental effects to the pulpal homeostasis and repair [8,17,20].

The amount of leachable components from composite resin networks has been found to be affected by the curing protocol and the density of crosslinking of the polymer network produced. However, a complex relationship exists between these two parameters and the extent that the elution process takes place. It is generally accepted that highly crosslinked polymers are more resistant to degradative processes, based on the more limited space and pathways available for solvent molecules to diffuse within the structure [23,64]. On the contrary, some other studies support that the higher the crosslinking density of a resin, the higher its heterogeneity and the larger the volume of micropores. This increase of the heterogeneity of the resin network enhances the elution process of the resin monomers [25,65]. These studies support that other factors, including the degree of conversion of the polymer and the quantity of pendant molecules existing within the network, may also affect the extent of water sorption and monomer elution.

Most studies also support that the elution from light-curing polymer-based materials is mainly influenced by the amount of energy delivered to the material during irradiation. The higher the energy density applied, the lower the elution into various solvents [46,66,67]. This can be explained by the increase in the degree of conversion with increasing energy density. However, for a given energy density, different combinations of curing time, power density and modes of cure (continuous, pulse-delay, or stepped) may significantly affect the elution process. Munksgaard *et al.* [46] observed that specimens cured with plasma arc for 3 s eluted a higher amount of monomers compared to specimens cured for 40 s with a conventional quartz–tungsten–halogen curing unit. Hofmann *et al.* [68] observed that different curing protocols influenced the solubility and water sorption of resin composites. Moon *et al.* [66] recorded different degrees of elution and softening in ethanol when a resin composite was cured with various curing units and curing protocols. Yap *et al.* [24] proved that with the same emitted energy level, the level of crosslinking of resin composites irradiated with continuous mode halogen curing is higher than LED-cured analogs. This is accompanied by more leached monomer and more pronounced toxic effects. Bennetti *et al.* [67] also found that the curing mode (continuous, step cured or pulse-delay mode) can significantly affect the crosslinking and degree of conversion of the material and therefore the process of elution. Therefore, it can be concluded from the above mentioned studies that the elution of elements and the degree of cytotoxicity of composite resins depends on the mode of polymerization process, including type of curing unit, total energy density, power density, irradiation time and mode of curing (continuous or different modes of soft start curing) [24,26,69].

## 3. Degradation of Resin–Based Dental Restorative Materials

As already mentioned, elution of substances from resin composites is usually completed within a few hours or days after initial polymerization. However, leachable substances may also be generated by erosion and degradation over time. The latter is of major biological significance, as it theoretically lasts as long as the service life of the material [6,16,22,54]. Resin degradation may be caused by photo, thermal, mechanical, or chemical influences. For example, it has been found that biologically derived enzymes, such as cholesterol esterase (CE) and pseudocholinesterase (PCE) can degrade the monomer components of composite resins, which may then result in the liberation of methacrylic substances [7,70]. Reviews of polymer degradation mechanisms have been already published [7,22]. Methacrylates degradation can produce different types of products through different mechanisms, such as formaldehyde via oxidation and methacrylic acid and other molecules, such as bis-HPPP, which is the dialcohol left after splitting methacrylic acid from bis-GMA by hydrolysis or esterification [23,41,71–74]. Other biodegradation products also include triethylene glycol methacrylate (TEGMA), 2,3–epoxymethacrylic acid (2,3-EMA) and ethoxylated bisphenol A (E-BPA) [75,76]. The biodegradation process mainly depends on the molecular chemistry. TEGDMA has been shown to be more susceptible to enzymatic hydrolysis than Bis-GMA or Bis-EMA [71,77]. Moreover, chemically modified BisGMA (ethoxylated BisGMA) degrades to a lesser degree in the presence of cholesterol esterase as compared to BisGMA. In addition, urethane modified bis-GMA/TEGDMA networks have been shown to be more stable in the presence of cholesterol esterase than unmodified bis-GMA/TEGDMA networks [74]. It is also important to note that not all esterases have demonstrated the same specificity for monomer components. Kinetic studies have shown that PCE preferentially hydrolyzes TEGDMA over BisGMA, while CE’s activity with respect to BisGMA is 14 times greater than that of PCE [73]. Therefore, it can be concluded that resinous matrix degradation, caused through different mechanisms is mainly dependent on the molecular chemistry of the monomers released, as well as the enzymatic activity of each individual.

Little is known however with respect to pharmacokinetics and toxicokinetics of degradation products of resin components. The existing studies support that HEMA and TEGDMA monomers when administered by different routes (oral, subcutaneously or intravenously) are almost completely eliminated 24 hours after administration. The main routes of excretion in animal studies are via the lungs and to a lesser extend via the faeces or the urine [78]. This implies that the concentration of these monomers in different tissues is below those known to cause acute toxic effects. However, several studies support that sub- cytotoxic concentrations of these monomers are able to alter cell function [79]. Further investigation is necessary to clarify the *in vivo* degradation and toxicokinetics of substances released by dental composite resins.

Another very important molecule from a biological point of view is Bisphenol A (BPA), due to its well documented estrogenic activity [80–82]. BPA is used in the production of several types of resins used in a variety of products including food and drink containers, CDs etc. The majority of published studies were not able to identify BPA as a degradation product of BisGMA-based composites, despite the fact that several of these studies used extreme elution conditions with respect to pH, organic solvents (*e.g.,* acetonitrile) and presence of different hydrolytic enzymes (esterases) [83–87]. On the other hand, BPA was found to be eluted as a degradation product of BisDMA, which is commonly found as a component of pit and fissure sealants [88–91]. The latter has been considered the main cause of their reported estrogenic effects [88,90]. In contrast, however to the abovementioned studies, Pulgar *et al.* [92] reported considerable release of BPA (up to 1.8 μg/mg of resin) and other related aromatic compounds with estrogenic effects (Bis-DMA, 1.15 pg/mg), bisphenol A diglycidylether (6.1 pg/mg), Bis-GMA (2.0 pg/mg) and ethoxylate and propoxylate of bisphenol A from Bis-GMA-based composites. These concentrations have found to be able to cause significant biologic effects in *in vivo* experimental models [80,93–98].

BPA has been also detected in the saliva and urine samples of healthy donors immediately after composite placement. Arenholt-Bindslev *et al.* [89] reported that minute amounts of BPA were detected in saliva samples collected immediately after, but not 1 h and 24 h after placement of dental sealants. Fung *et al.* [99] also analyzed the blood samples and saliva of a patient population and concluded that even if small amount of BPA was present in the saliva immediately after placement of the sealant, it could not be detected in their blood samples. Sasaki *et al.* [100] detected BPA using an ELISA system, in the range of several tens to 100 ng/mL in the saliva of healthy donors after filling teeth with two pit and fissure sealants. BPA was found however to be removed with sufficient gargling after treatment. Joscow *et al.* [101] found that BPA concentrations in saliva samples of healthy donors collected immediately after a BisDMA containing sealant placement were more than 50-fold higher than their baseline BPA concentrations, also in the range of several tens of ng/mL, whereas urinary concentrations one hour after placement were five times higher than their baseline levels. It can be concluded that even if BPA concentration is reduced after resin materials placement these results cause significant concerns with respect to the long term exposure to estrogenic substances released by composites, especially when it is added to the environmental exposure to several xenoestrogens.

## 4. Molecular Toxicology of Substances Released by Composite Resins

### 4.1. Cytotoxicity and Genotoxicity of Released Substances

The cytotoxicity and genotoxicity of substances released by dental composite resins has been extensively studied during the last two decades. Most studies have focused on the effects of resin compounds on basic cellular functions, such as cell proliferation, inhibition of enzyme activities, disruption of cell morphology, membrane integrity, cell metabolism (DNA-, RNA- and protein synthesis) and cell viability. These effects have been already reviewed by Geurtsen [16] and Schweikl *et al.* [20]. Most studies have shown that dental composite resins are able to release compounds with severe (Bis-GMA, UDMA, TEGDMA, DMBZ, και DMDTA) or medium (HEMA, BEMA, CQ, DMPT and DMAPE) cytotoxicity, whereas their biodegradation products, such as methacrylic acid, have been shown in general to be less cytotoxic [44,54,62,102,103]. The latter can also explain the fact that the cytotoxic effects of these compounds are reduced by the action of a metabolically active microsomal liver fraction (S9 mix) [104,105]. Among the substances released, the major (co)monomers have been identified as the main cause of cytotoxicity and their TC50 have been evaluated in a variety of cell culture systems, including permanent cell lines (3T3 and L929 fibroblasts, V79 chinese hamster lung fibroblasts, HaCaT keratinocytes, THP-1 monocytes etc), as well as primary cell lines of human origin (pulp, periodontal, gingival or skin fibroblasts), presenting significant variability in their sensitivity. Despite these differences, in most studies the cytotoxicity ranking of the basic monomers has been found to be the following: BisGMA > UDMA > TEGDMA >>> HEMA [54,102,104,106,110–113]. Moreover, a relationship between the structural and biological activities of the monomers has been reported [114].

### 4.2. Molecular Mechanisms

Taking a step forward, a considerably growing number of studies has been focused on the investigation of the key molecular mechanisms and signaling pathways involved in resin components-induced cytotoxicity and genotoxicity. These mechanisms have been already reviewed by Schweikl *et al.* three years ago [20]. However, considerable scientific knowledge during the last few years has been added with respect to the molecular toxicology of these substances. The studies relevant to the molecular mechanisms underlying the resin components’ induced cytotoxicity and genotoxicity are presented in Tables 1 and 2 respectively, mainly focusing on the studies of the last decade, that have been conducted in target tissues of the oral cavity.

#### 4.2.1. TEGDMA (Triethyleneglycol Dimethacrylate)

TEGDMA has been the most extensively studied resinous monomer with respect to biocompatibility, since it is easily released from polymerized composites into aqueous media and accounts for most of the unreacted double bonds [23,42]. Moreover, TEGDMA is a commonly used diluent of many resin-based dental composites and also a common component of dentin adhesives in contents varying from 25 to 50% [2,6]. Due to its lipophilic nature, TEGDMA can easily penetrate the cytosol and membrane lipid compartments of mammalian cells [115].

TEGDMA has been reported to induce time- and concentration- dependent cytotoxicity in various cell lines, as shown in Table 1. In most studies, TEGDMA concentration ranged from 0.5–5 mM. Moreover, its lethal concentrations have been reported to vary in different cell lines and among the same types of cells obtained from different donors [116,117]. It is also to note that some of the metabolic products of TEGDMA, such as the epoxy compound 2,3-epoxymethacrylic acid (2,3-EMA) have been found to cause comparable cytotoxic effects, contributing to TEGMA cytotoxicity. On the other hand, other metabolites, such as triethylene glycol (TEG and methacrylic acid (MAA) have shown minimal cytotoxicity [118]. At lower concentrations the predominant type of cell death induced by TEGDMA was apoptosis (programmed cell death), whereas necrosis was more pronounced at higher concentrations [109,119–121]. TEGDMA–induced apoptosis was enhanced by its inhibitory effect on phosphatidylinositol 3-kinase in primary human pulp cells [116] and by differential activation of MAP-kinase signaling pathways [121,122]. There is evidence that the balance between the sustained activation of the MAP kinases ERK1/2 and the stress kinases p38 and JNK is most likely a central factor in the regulation of cell death and survival in TEGDMA-treated cell cultures [122].

TEGDMA-induced apoptosis was also found in a number of studies to be associated with oxidative stress via Reactive Oxygen Species (ROS) generation [121,123,124]. This was further supported by the fact that its cytotoxicity was reduced in the presence of antioxidants, such as *N*-acetylcysteine (NAC), ascorbate, vitamins A and E (Trolox), uric acid etc [123,125–127]. ROS generation was accompanied in various cell lines by depletion of intacellular glutathione (GSH), a major natural reducing agent implicated in cellular detoxification and maintenance of redox balance. [128–131]. Lefeuvre *et al.* [128] also found significant reduction of glutathione transferase P1 activity by TEGDMA in human gingival fibroblasts. They supported that TEGDMA is a non-competitive antagonist of GSTP1 and that GSTP1 polymorphism could be involved in inter-individual susceptibility to TEGDMA cytotoxicity. The same authors supported that GSH depletion was accompanied by lipid peroxidation and mitochondrial damage, indicated by a collapse of the mitochondrial membrane potential [132]. These effects were significantly reduced by a soluble derivative of tocoferol (vitamin E) and by CCCP (carbonylcyanide *m*-chlorophenylhydrazone), an uncoupler of oxidative phosphorylation on lipid peroxidation and LDH leakage.

Several studies have supported that the cell death pattern could be important regarding the evaluation of the potential of dental materials to cause adverse effects [110,120,133], as apoptotic cells are removed by phagocytosis and with little inflammatory response. The latter is in sharp contrast to the inflammation and injury to surrounding tissues induced by the necrotic process [134,135].

TEGDMA has been also reported to induce significant genotoxic damage at subtoxic concentrations. It has been found to increase the number of micronuclei [104,109] and promote degradation of DNA derived from salivary gland tissue and lymphocytes, as shown in comet assays [136,137]. The induction of micronuclei was however clearly abolished by a microsomal fraction (S9) from rat liver, which indicates that the metabolites of TEGDMA are not able to cause genotoxic damage. Antioxidants were also able to reduce TEGDMA induction of micronuclei [123]. TEGDMA was also reported to induce extensive deletions of nucleotide sequences in the hypoxanthine-guanidine phosphoribosyltransferase (*hprt*) gene in V79 Chinese hamster lung fibroblasts, which is indicative of the clastogenic potential of this chemical [138]. Most recently, it has been reported that TEGDMA is able to cause oxidative DNA damage, indicated by the generation of 8-oxoG, followed by activation of ATM, which by itself might activate pathways leading to apoptosis [122]. Moreover, Schweikl *et al.* [124] have shown using microarrays technology that TEGDMA-induced cell damage is followed by a coordinated induction of genes coding for significant biological processes, including oxidative stress, cellular growth, proliferation and morphology, cell death, DNA replication and repair. The most upregulated genes were GEM, KLHL24, DDIT4, TGIF, DUSP5 and ATF3, which are related to the regulation of the cell structure, stress response and cell proliferation, whereas the most down-regulated transcript was TXNIP which regulates the cellular redox balance. As a consequence of DNA damage, different patterns of cell cycle delays-mainly in G2 phase- have been reported for different cell lines exposed to TEGDMA, in order to allow DNA repair processes [139,140]. These delays have been shown to be mediated through both p53-dependent and p53-independent pathways, in different cell lines [140].

Of major clinical significance are the long term effects of TEGDMA at subtoxic concentrations. It has been reported that TEGDMA cannot induce TNF-a release from THP-1 monocytes by itself, but it suppresses LPS-induced TNF-a secretion, suggesting some modification of the normal inflammatory response of pulpal tissues [141]. Moreover, other inflammatory mediators, such as IL-6 and IL-8 are released from 3-D cultures of TR146 cells exposed to TEGDMA [142]. Most recently, it has been shown that TEGDMA modulates LPS-induced production of not only TNF-a, but also of many other cytokines. It has been found to suppress IL-6 and IL-10 production by about 90% and CD14 expression at high concentrations. Moreover, CD40 and CD80 were down-regulated, whereas CD86 and MHC class I were inhibited to a lesser extent. On the contrary, CD54 was increased about twofold by increasing TEGDMA concentrations [143]. TEGDMA has been also found to induce cytokine MCP-1 secretion from U937 cells and to increase the hydrolase activity in human gingival fibroblasts [144]. Other inflammation markers, including Prostaglandin E2 were found to be increased in murine macrophages [142]. Overall, these data suggest that TEGDMA has a strong influence on the interaction of immune cells, including presentation of antigens, co-stimulation of T-cells, and cell–cell interactions [145].

Long term exposure to subtoxic concentrations of TEGDMA is not only able to affect immune responses but also other physiological processes, such as wound healing, cell differentiation and cellular metabolism. It has been found that TEGDMA is able to affect the physiological differentiation processes of dental pulp fibroblasts into odontoblasts and their normal mineralization procedure at very low concentrations [79]. TEGDMA has been found to modulate stress response by suppressing the expression of heat shock proteins, such as HSP72 [62]. Moreover, in a very interesting study by Engelmann *et al.* [146] TEGDMA was detected by NMR spectroscopy in all cellular fractions (cytosol, lipid fractions, as well as the culture media) and was able to affect the metabolic state of the cells by increasing the ratio of nucleoside diphosphates to nucleoside triphosphates.

Therefore, it can be concluded from the above presented studies that TEGDMA is a very active methacrylate molecule, that is able to cause not only pronounced cytotoxic and genotoxic effects mainly through oxidative stress pathways in different cell types but also to influence significant cellular functions implicated in immune response, wound healing and cellular metabolism even at very low (subtoxic) concentrations.

#### 4.2.2. HEMA (2-Hydroxy-ethyl-methacrylate)

HEMA has been also widely studied for biocompatibility, as it is one of the most common components of dentin-adhesives, ranging from 30 to 55% and has a pivotal role during the dentin impregnation process of adhesive systems. This is due to its high water affinity, which allows HEMA to flow into the collagen network of the dentin organic matrix, thus favoring infiltration and preventing collagen collapse [147]. Because HEMA has a low molecular weight and high hydrophilicity, it can also diffuse throughout the residual dentin and affect the underlying odontoblast vitality, altering cell division and physiological activity [61,148]. According to Spagnuolo *et al*. [126] the release of HEMA from polymerized dental adhesives ranges from 1.5 mmol/L to 8 mmol/L.

In terms of cytotoxicity, HEMA has been found to be far less toxic, as compared to the bifunctional monomers [102,104,106–111,116]. However, the TC50 concentration varied significantly with different cell lines and among the same types of cells obtained from different donors, ranging from 3.6 mmol/L to 10 mmol/L in various studies [63,112,117,126]. According to most of these studies the cytotoxicity of HEMA was time-and concentration-dependent.

HEMA induced cytotoxicity was also associated with oxidative stress, indicated by ROS production and depletion of intracellular glutathione [111,130,131,149]. These effects were found to be reduced in the presence of antioxidants [109,125,126]. Chang *et al*. [149] however reported that ROS production induced by HEMA is probably not followed by GSH depletion in human gingival epithelial cells, because GSH depletion was marked only at high concentrations, while an excessive ROS production was noted also at lower concentrations. Likewise, a significant change of the GSH-GSSG ratio was not assessed in THP-1 human monocytic cells after treatment with HEMA sub-lethal concentrations [130].

The resulting imbalanced redox state caused by HEMA is further associated with cell cycle delays and apoptosis involving activation of caspases-8,-9 and -3 [139,149,150]. HEMA induced apoptosis was found to be associated with the activation of nuclear factor kappa B (NF-kB), which plays a protective role to counteract HEMA cytotoxicity [150] and differential MAP kinase activation, including phosphorylation of JNK and p38 [121]. HEMA induced apoptosis has been also proposed as an important mechanism for the generation and persistence of hypersensitivity reactions of patients to this monomer. Paranjpe *et al.* [151] have shown that HEMA induced a dose-dependent apoptosis in Peripheral Blood Mononuclear Cells (PBMCs) of both healthy and HEMA-sensitized patients. However, induction of cell death by HEMA was lower in PBMCs obtained from patients in comparison to healthy individuals. On the contrary, other studies with primary human gingival fibroblasts cultures have supported that HEMA induced cell death is mainly in the form of necrosis rather than apoptosis [126,152].

In terms of genotoxicity, HEMA has been also reported to be a clastogenic chemical by increasing the number of micronuclei, effects that were however diminished after metabolic inactivation [104,109]. It has been also found to increase DNA migration in Comet assays [136,137]. These effects were followed by cell cycle delays, but were found to be reduced in the presence of antioxidants [127].

Several studies have also evaluated the effects of HEMA at very low concentrations in long-term cytotoxicity systems that are more relevant to clinical conditions. HEMA has been found to alter the normal inflammatory response of pulpal tissues, by significantly reducing TNF-a secretion from LPS-stimulated human THP-1 monocytes and peripheral blood monocytes [108,141,145,153]. These findings are further supported by the fact that HEMA was found to induce up-regulation of COX-2 [145] and VEGF expression [154], as well as suppression of Hsp72 expression in immune cells [62], suggesting its implication in inflammation related processes caused by composite materials. Other long term effects of HEMA include the interruption of normal collagen I synthesis [117,148] and the significant perturbation of normal differentiation processes of pulp fibroblasts into odontoblasts [79], which has a critical significance in pulpal homeostasis and repair.

In conclusion, HEMA was also found to be a very active biologic molecule, although its cytotoxicity is much lower compared to the bifunctional monomers TEGDMA and BisGMA. However, its pivotal role during composites adhesion into dentin and its high mobility due to its hydrophilicity and low molecular weight make it a critical molecule from the viewpoint of biocompatibility. The mechanisms of its cytotoxic and genotoxic effects seem not to differ from those of TEGDMA and mainly involve oxidative stress via ROS production. Of significant importance are also the long term effects of HEMA at subtoxic concentrations, which are able to disturb physiological pulp homeostasis and repair.

#### 4.2.3. Basic Monomers BisGMA (2,2-Bis[4-(2- hydroxy-3-methacryloxypropoxy) phenyl]propane) and UDMA (Urethane dimethacrylate)

The basic bifunctional resinous monomers BisGMA (2,2-bis[4-(2-hydroxy-3-methacryl-oxypropoxy)phenyl]propane) and UDMA (urethane dimethacrylate) have been also studied for cytotoxicity and genotoxicity in a considerable number of studies. In general, the aromatic monomer BisGMA has been found to be slightly more cytotoxic than the aliphatic monomer UDMA [102,104,106–110,112,116,152]. Despite the fact that BisGMA is not readily soluble in water and available only in small amounts in a hydrophilic environment it has been used as a representative acrylate compound for studying the toxic mechanisms of resin monomers on biological tissues [155,156]. On the other hand, UDMA, that has been often used today to replace BisGMA in many commercially available dental composites due to its high flexibility and toughness, represents a family of molecules with different molecular weight and structure that have been relatively less studied compared to other methacrylate molecules [157].

BisGMA (>0.001 mM) and UDMA (0,05 mM) have been found to cause time- and concentration-dependent cytotoxicity to various cell lines, including human gingival and pulp fibroblasts and human THP-1 and peripheral blood monocytes [108,131,153,156,158,159]. Bis-GMA have been also found to induce a rapid and intense decline of the glutathione pool of HGFs combined with induction of apoptosis at much lower concentrations (>0.1 mM) as compared to TEGDMA (>5 mM) [156]. BisGMA could also stimulate ERK phosphorylation, PGE2 production, COX-2 mRNA and protein expression, as well as ROS production. Catalase and U0126 (a MEK inhibitor) were able to effectively prevent the above mentioned effects [155]. These findings suggest that BisGMA released from composite resins may potentially affect the vitality of dental pulp and/or induce pulpal inflammation. This is further supported by the fact that BisGMA is able to disturb normal differentiation procedures of pulp fibroblasts [79,160]. Other long term effects of BisGMA include its ability to affect the migration and tenascin expression of keratinocytes and human gingival fibroblasts, possibly disturbing the healing of injured oral tissues [161]. Moreover, BisGMA and its biodegradation product methacrylic acid (MMA) have been found to significantly decrease ICAM-1 expression in TNF-a-stimulated cells, which suggests that these methacrylates may decrease the recruitment of leukocytes towards the inflammation sites [158].

Concerning genotoxicity, BisGMA and UDMA has been also found to increase the number of micronuclei and these effects to be reduced by S9 mix, in the same way as with TEGDMA and HEMA [104]. DNA migration has been also reported in Comet assays for these monomers [136,137]. On the other hand, the hydroxylized metabolites of Bis-GMA, such as Bisphenol A bis (2,3-dihydroxypropyl) were found to be non-mutagenic and less cytotoxic than their parent monomer [159].

In conclusion, the basic resinous monomers BisGMA and UDMA, which account for about 70-75% of the total resinous matrix of dental composites may significantly contribute to these materials cytotoxicity and genotoxicity. Despite their hydrophobic character which limits their release into aqueous environments they are able to exert their cytotoxic action at much lower concentrations as compared to HEMA and TEGDMA. Involved mechanisms seem also to include oxidative stress, as well as disturbance of normal biological processes, such as differentiation, immune response and wound healing at very low concentrations.

### 4.3. Compounds of Dental Composites’ Polymerization System

Extractable components of resin-based dental restorative materials also include *substances of their polymerization system*, such as photosensitizers and initiators [16]. Camphoroquinone (CQ) is the most commonly used photosensitizer and has been found to be eluted by various resin composites. Very few studies up to now have addressed the potential biological adverse effects of CQ. It has been shown that CQ in the presence or absence of reducing agents was cytotoxic to a human submandibular duct cell line, as well as to human gingival and pulp fibroblasts [162,163]. In addition, many of the most known polymerization initiators, such as CQ, benzoyl peroxide (BPO) dimethylaminoethyl methacrylate (DMAEMA) and dimethyl-*para*-tolouidine (DMPT) have been found to be cytotoxic to human gingival fibroblasts by inducing cell cycle arrest and cell death mainly in the form of necrosis [164]. When compared to other photosensitizers, such as benzil (BZ), benzophenone (BP), 9-fluorenone (9-F), CQ was found to be less cytotoxic and to produce less ROS. Moreover, ROS induced by the aliphatic ketone CQ were efficiently scavenged by hydroquinone and vitamin E, whereas those by the aromatic ketone 9-F were diminished by mannitol and catalase, suggesting that OH radicals were involved in ROS derived from 9-F [162]. In addition, CQ in combination with visible light (VL) irradiation was found to increase the radical production, whereas 9F with VL irradiation increased ROS production and effecting changes in the phase-transition properties of DPPC liposomes, which were used as a model for cell membranes. The addition of DMA (a tertiary amine) to the photosensitizer enhanced the free-radical production without increasing the ROS level or the cytotoxicity. The authors concluded that CQ/DMA is a valuable combination for the polymerization of dental resins because of its less photo-oxygenation and cytotoxicity together with its great ability to cause polymerization of methacrylates. On the other hand, another scientific group has shown that CQ/DMT with or without VL irradiation was able to cause significant prolongation of the cell cycle. In addition, VL irradiated CQ/DMT was found to exhibit significantly genotoxic and cytotoxic effects, compared with CQ/DMT alone. These effects were however reduced by pre-treatment with antioxidants [165]. These results are in agreement with Pagoria *et al.* [166] who reported that VL irradiated CQ/DMT caused DNA strand breakages in isolated supercoiled plasmid DNA, and Winter *et al.* [167], who demonstrated that VL irradiated CQ/DMT caused DNA damage in a cell-free environment. Moreover, recently, Pagoria and Geurtsen [168] have published that VL irradiated CQ/DMT caused oxidative damage in 3T3-Swiss albino murine fibroblasts and murine cementoblasts. They also confirmed the protective effect of high concentrations of NAC (10 mM) and ascorbic acid (10mM) in these cell lines. Taken together, these results suggest that the CQ/DMT system can act as a genotoxic agent.

Other substances of the polymerization system of dental composites have been also studied to a lesser extent for biocompatibility. Cimpan *et al.* [169] have found that 4-*N,N*-dimethylaminobenzoic acid ethyl ester (DMABEE), one of the compounds commonly being eluted, was able to cause time- and concentration- dependent induction of cell death in human monoblastoid cells in the form of apoptosis and necrosis. Other studies reported that DMABEE is also able to interact with monolayers of saturated phosphatidylcholines (PC, *i.e.,* markers of the outer membrane leaflet) and phosphatidylserines (PS., *i.e.,* markers of the inner membrane leaflet) [170].

In conclusion, several compounds eluted from dental composites’ polymerization system are able to significantly contribute to their cytotoxicity and genotoxicity by enhancing the oxidative stress and DNA damage. These effects are significantly increased by visible light irradiation of these systems.

### 4.4. Effects of Composite Resins’ Compounds on Oral Bacteria Growth

Most studies support that pulp inflammation caused by derivatives of resin composites is mainly due to incomplete dentin adhesion, which leads to bacterial microleakage [171–173]. However, there are also some studies supporting that dental monomers, such as TEGDMA and EGDMA are able to promote the growth and proliferation of caries relevant bacteria, such as *S. Sobrinus* και *L. Acidophilus* [174,175] and by this way to contribute to pulpal inflammation and secondary caries formation. Kawai *et al.* supported that these monomers are not only able to increase bacterial growth but also to increase glycosyltransferase activity which is responsible for glycanes formation that play a key role in bacterial adhesion and plaque formation. Moreover, Khalichi *et al.* [176] supported that several by-products of TEGDMA, such as TEG, are also able to increase glycosyltransferase B expression in *S. mutans.* On the contrary, Takahasi *et al.* [177] claimed that ethyleneglycol monomers do not increase in fact microbial proliferation but the observed biomass increase is mainly due to polymerization of resin monomers to form vesicular structures attached to cells.

There has also been and effort to produce resin monomers with antibacterial properties, such as MDPB (methacryloxydodecylpyridinium bromide) or composites fillers based on apatite and contain silver and zinc (ApaciderTM ή Novaron) [178–180]. Although these substances are able to reduce the proliferation of cariogenic bacteria including *Str. Mutans,* they are usually immobilized by polymerization and therefore diffusion through the dentin is no longer possible [179].

## 5. Discussion and Conclusions

Studies on the molecular toxicology of substances released by resin-based dental restorative materials clearly support that the majority of these molecules are able to cause cytotoxic and genotoxic effects at concentrations relevant to those released into the oral cavity. These effects include irreversible disturbance of basic cellular functions, such as cell proliferation, enzyme activities, cell morphology, membrane integrity, cell metabolism and cell viability. Signaling pathways involved in immune response, tissue homeostasis and repair are also affected. Moreover, several studies have reported the clastogenic and genotoxic properties of some of these substances, implying their potential mutagenic effects and stressing the importance of assessing their safety from the viewpoint of genotoxicity.

The clinical relevance of identifying the potential of these substances to disturb functions at the cellular and molecular level has been already emphasized by experienced investigators in the field [20,181]. However, the direct extrapolation of molecular toxicological data obtained from *in vitro* studies into the clinical situation is not always straight forward. At the local level, a large number of *in vivo* studies with animal or human teeth (usage tests) support that pulp reaction is not expected in medium or low depth cavities, when a sufficient thickness of dentin layer remains and bacterial penetration beneath the filling is avoided [182–185]. On the other hand, other studies support that there are pronounced histological reactions when the remaining dentin is too thin and acid etched [186,187]. The same detrimental effects, including pulp inflammation, insufficient reparative dentin formation and even pulp necrosis are also reported when resin adhesives are used for direct pulp capping instead of calcium hydroxide [188–191]. Further research on this aspect is necessary.

The clinical significance of *in vitro* mutagenicity and genotoxicity data is also quite difficult to be assessed, since no information is up to now available concerning the threshold concentrations that are able to trigger these reactions during the long term clinical service of these materials. Moreover, the toxicokinetics of the metabolic products of dental composites and the possibility for systemic mutagenic effects should be further investigated in animal models.

Although the frequency of adverse effects caused by resin based dental restorative materials, mainly allergic reactions in patients and dental personnel [192–195], has increased over the past years, the total number of patients presenting with adverse reactions still remains a low proportion of the total population. However, despite the fact that general risk seems to be quite low, the individual health risk during the long term clinical service of these materials, attributed to interindividual variations in immune responses and reparative processes, cannot be underestimated, especially in severe allergic cases [19].

It is surprising however that despite the rapidly growing bulk of scientific evidence concerning the toxicological effects of these substances, little effort has been observed form the part of the companies to develop new materials not only with improved mechanical but also biological properties. The majority of commercially available products are based on methacrylate monomers, whereas some promising new technologies, including Siloranes and Ormocers [196,197], using different chemistry and polymerization mechanisms are yet to be investigated from the viewpoint of biocompatibility. Taking into account that dental composite resins have an integral role in every day dental clinical practice, it is extremely important to encourage not only the development of less cytotoxic materials but also, as a future goal, the development of “biomimetic” materials or “biofillings”, which will be effective in stimulating natural tissue repair and maintaining the vitality of the compromised oral tissues.

## Figures and Tables

**Table 1.** Mechanisms of cytotoxic effects of substances released by resin-based dental restorative materials.

**Study**	**Substances studied (concentration)**	**Cell line**	**Biological parameters assessed**	**Methods**	**Main conclusions**
Geurtsen *et al.* 1998 [116]	35 resin composite monomers/additives	Human primary fibroblasts from attached gingiva (HGF) pulp (HPF) and the periodontal ligament (HPLF) and 3T3 swiss albino mouse fibroblasts	1. Cytotoxicity	1. Spectrophotometrically (Hoechst 33342)	Within the tested compounds, severe (*e.g.,* Bis- GMA, UDMA, DMBZ, and DMDTA, BHT, HMBP) or moderate (HEMA, BEMA, CQ, DMPT, and DMAPE) cytotoxic effects could be recorded. Reaction/decomposition products displayed only slight effects.
Rakich *et al.* 1999 [153]	Bis-GMA, UDMA, HEMA, 4-META	Human THP-1 monocytic cells	1. Cytotoxicity 2. Secretion of II-1b &TNF-a (±LPS)	1. MTT 2. ELISA	All monomers decreased LPS-induced release of TNFa & IL1-b at TC50 concentrations.
Li *et al.* 1999 [139]	HEMA	Human Pulp Fibroblasts (HPF)	1. Cytotoxicity 2. Cell cycle	1. MTT 2. FACs (PI)	HEMA induced dose dependent cytotoxicity and cell cycle arrest in G2 phase.
Bouillaguet *et al.* 2000 [198]	HEMA	Human THP-1 monocytic cells	1. Cytotoxicity 2. Protein synthesis	A. Trypan Blue assay B. BCA assay	HEMA significantly reduced cell proliferation but increased mitochondrial activity and protein synthesis after long term exposure to subtoxic concentrations (0.75 mM)
Theilig *et al.* 2000 [161]	TEGDMA (0.25–5 mM) BisGMA (0.001–0.1 mM)	Human gingival fibroblasts (HGFs) and HaCaT (human keratinocytes)	1. Cell Proliferation 2. Cell Migration 3. Tenascin expression	1. DNA synthesis (BrdU incorporation) 2. Modified boyden chamber assay 3. Immunocytochemistry and spectrophotometry	Proliferation of both cell types was significantly inhibited at concentrations >0.25 mM (TEGDMA) or > 0.01 mM (BisGMA). BisGMA (at 0.01 mM) but not TEGDMA significantly affected migration of keratinocytes and altered the expression of tenascin of HGF and HaCaT cultures. Thus, BisGMA may influence the healing of injured oral tissues.
Noda *et al.* 2002 [62]	HEMA (0–40 mM) TEGDMA (0—3 mM)	Human THP-1 monocytic cells	1. Cytotoxicity 2. Heat Shock protein 72 expression	1. MTT 2. SDS-PAGE Gel Electrophoresis & Immunoblotting	HEMA and TEGDMA significantly suppressed heat induced HSP72 expression, even at sublethal levels, but did not induce HSP72 by themselves. These results suggest that these monomers could modulate the HSP stress response without altering cellular metabolic activity.
Engelmann *et al.* 2001 [146]	TEGDMA (0.5 mM) HMBP (0.05mM)	Mouse 3T3-fibroblasts	1. Metabolic effects	1. NMR spectroscopy	TEGDMA could be detected in all fractions (cytosol, lipid fractions and culture media) of 3T3 cells, while HMBP was found only in the lipid fraction. Additionally, TEGDMA changed the metabolic state of cells, indicated by slight decreases of nucleoside triphosphates and an increase in the ratio of nucleoside diphosphates to nucleoside triphosphates
Kostoryz *et al.* 2001 [159]	BisGMA (0–50 μM) MAA (0–1,200 μM) CyracureTM UVR 6105 (epoxy monomer) (0–800 μM)	Endothelial cells, ECV 304 (TNF-a stimulated)	1. Cytotoxicity 2. ICAM-1 expression	1. MTT 2. FACs (anti–ICAM-1 antibody)	Except for UVR 6105, the methacrylates significantly decreased ICAM-1 expression in TNF-a-stimulated cells, which suggest that methacrylates may decrease the recruitment of leukocytes to inflammation sites.
Atsumi *et al.* 2001 [162]	CQ, BZ, BP, 9-F +DMT	1. Cell free system 2. Human gingival fibroblast (HGFs) and a human epidermoid carcinoma cell line from a sub-mandibular gland tumor.	1. Production of free radicals 2. Cytotoxicity	1. Spectrophotometrically (reduction of DPPH) and from the conversion of TEGDMA to polymers 2. MTT	The cytotoxic effects of the photosensitizers studied decreased as follows: CQ\BP\9-F\BZ. ROS production was dose- and time- dependent, and declined in the order: BZ\9-F\ BP\ CQ. ROS induced by aliphatic ketones (CQ) were efficiently scavenged by hydroquinone and vitamin E, whereas those by aromatic ketones (9-F) by mannitol and catalase, suggesting that OH radicals were involved in ROS derived from 9-F.
Heil *et al.* 2002 [108]	HEMA (10–300 mM) TEGDMA (0.5–10 mM) BisGMA (0.1–1 mM)	THP-1 monocytes Primary peripheral blood monocytes (PBM)	1. Cytotoxicity 2. TNF-a secretion with LPS stimulation	1. MTT 2. Spectrophotometrically	PBMs were 3–25 times less sensitive than TPH 1 cells but the cytotoxicity ranking of the components was identical BisGMA > TEGDMA > HEMA)
About *et al.* 2002 [79]	UDMA (1 μM) TEGDMA (10 μM) HEMA (10 μM) BisGMA (1 μM)	Human Pulp Fibroblasts (HPF)	1. Differentiation using culture medium containing β-glycophosphate	1. Histochemistry (alkaline phosphatase) and Immunocytochemistry (anti-collagen I, anti-dentin sialoprotein-DSP)	All monomers significantly decreased DSP expression and completely inhibited the normal mineralization process of HPCs expressed by mineral nodule formation. These effects were observed at nontoxic concentrations and were more pronounced for HEMA and BisGMA.
Janke *at al.* 2003 [119]	TEGDMA (1–7.5 mM)	Human gingival fibroblasts (HGFs)	1. Cytotoxicity 2. Apoptosis	1. Spectrophotometrically (Hoechst 33342) 2. FACs (Annexin V-PI) and microscopically	TEGDMA was cytotoxic and “apoptotic” in a dose- and time-dependent manner. TEGDMA at 5 and 7.5 mM inhibited proliferation and caused apoptosis, whereas no apoptosis or necrosis was observed with 1 mM or 2.5 mM TEGDMA.
Stanislawski *et al.* 2003 [123]	TEGDMA (0–3 mM)	Human gingival fibroblasts (HGFs) and Human Pulp Fibroblasts (HPF)	1. Cytotoxicity 2. GSH levers 3. ROS generation	1. MTT 2. Spectrophotometrically (mBCl) 3. Spectrophotometrically (DCFH-DA)	TEGDMA-induced cytotoxicity on HGFs and HPFs is associated with a rapid and drastic depletion of GSH followed by a production of ROS. Antioxidants, such as NAC, ascorbate, and Trolox, restored GSH levels to normal and appear to have a crucial role in cell protection.
Noda *et al.* 2003 [141]	HEMA (0–1.2 mM) TEGDMA (0—0.75 mM)	Human THP-1 monocytic cells	1. TNF-a secretion	1. ELISA	TEGDMA and HEMA did not induce TNF-a secretion by themselves, but significantly suppressed (40–70%) LPS induced TNF-a secretion at subtoxic concentrations.
Walther *et al.* 2004 [125]	HEMA (0.1–15 mM) TEGDMA (0.01–2 mM) in the presence of several vitamins (A, C, E, uric acid)	11Lu cells & 16Lu cells (human, lung, fibroblast-like), A549 (human lung cell carcinoma) and L2 cells (rat, alveolar epithelial)	1. GSH content 2. GSSG reductase activity 3. Protein determination	1,2 Spectrophotometrically (DTNB and NADPH) 3.methionine incorporation	All antioxidative substances were able to diminish the acute toxic effects of the monomers. 500 μmol/L Vitamin C or 250 μmol/L Vitamin E were mostly able to decrease toxicity of HEMA and TEGDMA in the cell lines tested.
Spagnuolo *et al.* 2004 [150]	HEMA (0–10 mM)	Human skin fibroblasts (HSF)	1. Apoptosis 2. ROS generation 3. NFkB expression	1. FACs (Annexin V-PI) 2. FACs (DCFH-DA) 3. SDS-PAGE, WB & EMSA	HEMA induced apoptosis in HSFs, involving activation of caspase-8,-9 and -3. Apoptosis was not directly dependent on the generation of ROS, as it was not reduced by antioxidants. Moreover, NF-kB plays a major role in protecting cells from HEMA induced apoptosis.
Spagnuolo *et al.* 2004 [120]	TEGDMA (0–3 mM)	Human Pulp Fibroblasts (HPF)	1. Apoptosis 2. PI3K Signaling	1. FACs (Annexin V-PI) 2. WB	Apoptotic and necrotic cell populations differentially increased after exposure to increasing concentrations of TEGDMA. A two-fold increase in the percentage of where apoptotic cells was induced by 1 mmol/L TEGDMA, as necrosis was more pronounced at 2 mmol/L. Inhibition of the MAP Kinase/ERK pathway had no influence on cell survival, but inhibition of PI3-Kinase amplified TEGDMA-induced apoptosis.
Lefeuvre *et al.* 2004 [128]	TEGDMA (0–3mM)	Human gingival fibroblasts (HGFs)	1. Cytotoxicity 2. GSTP1(glutathione transferase P1) genotyping, GSH, GSSG (oxidized GSH) levels and GSTP1 activity	1. MTT 2. Spectrophotometrically (various assays)	TEGDMA induces depletion of GSH and modulates the GSTP1 activity in both HGFs and a cell-free system. This is significantly more marked in the wild-type enzyme compared with the mutant one. Moreover, TEGDMA is a non-competitive antagonist of GSTP1. These data suggest that GSTP1 polymorphism could be involved in inter-individual susceptibility to TEGDMA.
Engelmann *et al.* 2004 [156]	Bis-GMA (0.001–0.25 mM)	Human gingival fibroblasts (HGFs)	1. Cytotoxicity 2. GSH content 3. Cell death	1. Spectrophotometrically (Hoechst 33342) 2. Spectrophotometrically (MBBr assay) 3. FACs (Annexin V/PI)	Bis-GMA induced a rapid and intense decline of the glutathione pool of HGFs combined with apoptosis at much lower concentrations (>0,1 mM) compared to TEGDMA (>5 mM)
Atsumi *et al.* 2004 [163]	CQ, 9-F +DMA (catalysts) (0.01–10 mM)	Human Pulp Fibroblasts (HPF)	1. Cytotoxicity 2. ROS production 3. phase-transition properties of dipalmitoylphosphatidyl choline (DPPC)	1. MTT 2. FACs (CDFH-DA, DCFH-DA) 3. differential scanning calorimetry	Camphoroquinone with VL irradiation increased the radical production, whereas 9F+VL irradiation increased ROS production, as well as effecting changes in the DPPC phase-transition properties. The cytotoxicity of CQ in HPF cells was smaller than that of 9F. The addition of DMA to the photosensitizer enhanced the free-radical production without increasing the ROS level or the cytotoxicity.
Lefeuvre *et al.* 2005 [132]	TEGDMA (0–3 mM)	Human gingival fibroblasts (HGFs)	1. Cytotoxicity 2. Oxidative stress 3. Mitochndrial damage 4. Lipid peroxidation 5. Mitochondrial membrane potential (MMP)	1. LDH determination 2. GSH determination 3. ATP determination (spectrofluorometrically) 4. TBARS determination 5. spectrofluorometrically (Rhodamine 123)	TEGDMA induced an increase of lipid peroxidation associated with LDH leakage and damage at mitochondrial level, demonstrated by the collapse of MMP of HGF. The effects were reduced by CCCP, an uncoupler of oxidative phosphorylation on lipid peroxidation and LDH leakage. Trolox, a soluble derivative of Tocopherol, weakly prevents ATP but not GSH depletion and totally protects the cells against lipid peroxidation, MMP collapse and cell death.
Paranjpe *et al.* 2005 [151]	HEMA (1.64–16.4 mM)	Peripheral Blood Mononuclear Cells (PBMCs) from both healthy and HEMA-sensitized patients & murine RAW cells	1. Apoptosis	1. FACs (Annexin V-PI) and TUNEL assay	HEMA induced a dose-dependent apoptosis in PBMCs of both healthy and HEMA-sensitized patients and in the RAW cells. However, induction of cell death by HEMA was lower in PBMCs obtained from patients in comparison with healthy individuals. This might be an important mechanism for the generation and persistence of hypersensitivity reactions in patients.
Engelmann *et al.* 2005 [129]	TEGDMA (0.1–5 mM) CQ (0.1–5 mM)	Human Pulp Fibroblasts (HPF)	1. ROS detection 2. GSH content	1. spectrophotometrically (DCFH-DA) 2. spectrophotometrically (MBBr assay)	TEGDMA significantly decreased GSH at concentrations between 0.5 and 5 mM but did not elevate ROS levels. Contrary, CQ increased ROS formation at concentrations > 1 mM, but had only a moderate effect on GSH at the highest test concentration.
Noda *et al.* 2005 [130]	HEMA (0–40 mmol/L) TEGDMA (0–3 mmol/L) BP (0–100 μmol/L) CQ (0–2 mmol/L)	Human THP-1 monocytic cells	1. Cytotxicity 2. GSH levels and GSH-GSSG balance	1. MTT 2. spectrophotometrically (Ellman’s method)	The results indicate that these dental resin compounds act at least partly via oxidative stress by increasing GSH levels at sublethal concentrations. However, the GSH-GSSG ratio was relatively unaffected.
Chang *et al.* 2005 [149]	HEMA (0.01–10 mM)	Human Pulp Fibroblasts (HPF) & human gingival epithelial Smulow – Glickman (S–G) cells	1. Cytotoxicity 2. Cell cycle 3. GSH depletion 4. ROS generation	1. MTT 2. FACs (PI) 3. FACs (CMF-DA) 4. FACs (DCFH-DA)	HEMA produced growth inhibition of HPF and S–G cells in a dose-dependent manner, accompanied by induction of GSH depletion, ROS production, cell cycle perturbation and apoptosis.
Schweikl *et al.* 2005 [140]	TEGDMA (0–3 mM)	V79 Chinese hamster lung fibroblasts (p53 deficient) N1 human skin fibroblasts (p53 proficient) Human Pulp Fibroblasts (HPF) (p53 proficient)	1. Cell viability 2. Cell cycle	1. Hemocytometer 2. FACs (PI)	TEGDMA caused different patterns of cell cycle delays in the three tested cell lines which were mediated both through p53-dependent (N1 fibroblasts and primary human pulp cells) and p53-independent (V79 cells) pathways.
Pagoria *et al.* 2005 [168]	CQ/DMT (1–2 mM)	3T3-Swiss albino murine fibroblasts (3T3) and Immortalized Murine cementoblasts (OCCM.30)	1. Oxidative stress after visble light irradiation of the CQ/DMT complex	1. spectrophotometrically (DCFH-DA)	VL-irradiated CQ/DMT produced significantly elevated intracellular oxidative levels in both cell types. OCCM.30 cells were found to bet twice as sensitive to VL-irradiated CQ/DMT compared to 3T3 cells. Furthermore, 10mM NAC and 10mM ascorbic acid were able to eliminate the oxidative stress.
Cimpan *et al.* 2005 [169]	DMABEE (0–200 μM)	U-937 monocytes	1. Cell death	1. FACs (Annexin V-PI)	DMABEE caused time- and concentration- dependent induction of cell death in the form of apoptosis and necrosis.
Spagnuolo *et al.* 2006 [126]	HEMA (0–12 mM) in the presence of the antioxidant NAC (1, 5, and 10 mM)	Human primary gingival fibroblasts (HGF)	1. Cytotoxicity 2. Cell Viability 3. ROS generation	1. MTT 2. FACs (Annexin V-PI) 3. FACs (DCFH-DA)	HEMA at concentration >10mM caused a decrease of cell viability, mitochondrial activity, and an increase of cell death. High NAC conc. (5, 10 mM) protect HGF against HEMA cytotoxicity by reducing the induced ROS levels.
Volk *et al.* 2006 [131]	HEMA (0.1–10 mM) TEGDMA (0.05–2.5 mM) UDMA (0.005–0.5 mM)	Human gingival fibroblasts (HGFs)	1. GSH content	1.spectrophotometrically (MBBr assay)	GSH depletion was dependent on the type of the resin monomer: UDMA > TEGDMA > HEMA.
Becher *et al.* 2006 [110]	HEMA (10–2,000 μgr/mL) TEGDMA (10–2,000 μgr/m;) GDMA (50–100 μgr/mL)	Primary alveolar mouse macrophages J774A1 mouse macrophages	1. Cytotoxicity 2. Apoptosis	1. MTT 2. FACs (Hoechst 33342) & Fluorescent microscopy (Hoechst 33342/PI)	The monomers’ cytotoxicity decreased as follows GDMA > TEGDMA > HEMA. The latter caused a greater accumulation of apoptotic cells
Reichl *et al.* 2006 [152]	HEMA (0.1–30 mM), TEGDMA (0.03–10 mM), BisGMA (0.01–0.3 mM), UDMA (0.01–1 mM)	Human gingival fibroblasts (HGFs)	1. Cytotoxicity 2. Cell death	1. XTT 2. Hoechst 33342 staining	The cytotoxicity of the monomers increased as follows: HEMA < TEGDMA < UDMA < BisGMA. TEGDMA induced mainly apoptosis, whereas HEMA, UDMA & BisGMA mainly necrosis.
Mantellini *et al.* 2006 [154]	HEMA, adhesives	Murine MDPC-23 odontoblasts, un-differentiated pulp cells (OD-21), HGFs and murine macrophages (Raw 264.7)	1. Cytotoxicity 2. VEGF expression	1. Trypan Blue B. ELISA	HEMA induced increased expression of VEGF only in MDPC-23 and Raw 264.7 cells. It seems that VEGF is implicated in angiogenesis in sites of pulp exposure that come in contact with dental adhesives.
Falconi *et al.* 2007 [117]	HEMA (1–10 mM)	Human gingival fibroblasts (HGFs)	1. Cell viability 2. Cell morphology, 3. Collagen I	1. MTT 2. SEM 3. Immunofluorescence	3 mmol/L HEMA did not induce cell death but caused a modification in the morphology of HGFs and a decrease in the type I collagen expression.
Moharamzad eh *et al.* 2007 [112]	BisGMA (0.02–10 mM) UDMA (0.02–10 mM) TEGDMA (0.02–10 mM)	Human gingival fibroblasts (HGFs) and HaCaT keratinocytes	1. Cytotoxicity 2. Inflammatory marker (IL-1β)	1. Alamar Blue assay 2. ELISA	Monomers were toxic to HGFs and HaCaT. The cytotoxicity ranking was BisGMA > UDMA > TEGDMA. However, they cannot induce IL-1β release from these cells by themselves.
Samuelsen *et al.* 2007 [121]	HEMA (0–15 mM) TEGDMA (0–3 mM)	Rat submandibular salivary gland acinar cells, SM 10–12	1. ROS generation 2. Cell death 3. Protein analysis of p-ERK p-JNK and p-p38	1. spectrophotometrically (DCFH-DA) 2. Hoechst 33342 staining 3. WB	HEMA or TEGDMA exposure resulted in ROS formation, concentration-dependent apoptosis and phosphorylation of ERK. Phosphorylation of JNK and p38 was induced by HEMA. Therefore, differential MAP kinase activation appears to be involved in HEMA and TEGDMA induced apoptosis.
Volk *et al.* 2007 [200]	TEGDMA (0–5 mM)	Human gingival fibroblasts (HGFs)	1. GSH content	1.spectrophotometrically (MBBr assay)	TEGDMA induced an early and drastic depletion of GSH that was more pronounced in the presence of H_2_O_2_.
Spagnuolo *et al.* 2008 [199]	HEMA (1–14 mM)	Human Pulp Fibroblasts (HPF)	1. Cell viability 2. ROS generation 3. Expression of P-Akt and P-ERK1/2	1. FACs (Annexin V-PI) 2. FACs (DCFH-DA) 3. WB	HEMA exposure modulated ERK and Akt pathways in different manners and these in turn function in parallel to mediate pro-survival signaling in HPF subjected to HEMA cytotoxicity.
Teti *et al.* 2008 [148]	HEMA (3 mM)	Human gingival fibroblasts (HGFs)	1. Cytotoxicity 2. Expression of pro-collagen a1.	1. MTT 2. Real Time RCR, WB and Immunofluorescence	Exposure of HGFs in 3 mM HEMA interferes both with the synthesis of the procollagen a1 type I protein and its mRNA expression, suggesting that normal cell production and activity are modified by HEMA at concentrations below those which cause acute cytotoxicity.
Reichl *et al.* 2008 [111]	HEMA (0.1–30 mM) TEGDMA (0.03–10 mM); BisGMA (0.01–0.3 mM); UDMA (0.01–1 mM)	Human gingival fibroblasts (HGFs) and Human Pulp Fibroblasts(HPF)	1. GSH content	1,2 Spectrophotometrically (DTNB and NADPH) 3.methionine incorporation	The addition of H_2_O_2_ (0.06 or 0.1 mmol/L) resulted in a toxicity potentiation of TEGDMA and UDMA, but not of HEMA and BisGMA, on HGF or HPF.
Emmler *et al.* 2008 [118]	TEGDMA (0.003–10 mM) TEG +MA (equimolar TEGDMA) (0.03–10 mM), TEG (0.03–10 mM) MA (0.03–30 mM) 2,3-EMA (0.001–30 mM) and PFA (0.03–10 mM).	Human bronchoalveolar carcinoma-derived A549 cells	1. Cytotoxicity	1. XTT	The epoxy compound 2,3-EMA induced comparable toxic effects as the raw TEGDMA. On the contrary, no cytotoxic effects could be found for TEG up to a concentration of 10mM. It was concluded that some toxic intermediates might significantly contribute to TEGDMA-induced cytotoxicity.
Schweikl *et al.* 2008 [124]	TEGDMA (1 mM and 3 mM)	Normal human skin fibroblasts (N1)	1. ROS generation 2. Cell cycle analysis 3 Gene expression analysis	1. FACs (DCFH-DA) 2. FACs (PI)	TEGDMA at 3 mM increased ROS production and caused a cell cycle delay after 6 hours. The predominant biological processes associated with the genes that were differentially expressed included oxidative stress, cellular growth, proliferation and morphology, cell death, DNA replication and repair. The most upregulated genes were GEM (17-fold), KLHL24, DDIT4, TGIF, DUSP5 and ATF3, which are related to the regulation of the cell structure, stress response and cell proliferation. TXNIP was the most downregulated transcript, which regulates the cellular redox balance.
Gregson *et al.* 2008 [144]	TEGDMA (1.25 and 1.5 mM)	Monocyte derived macrophage (U937) cells Human gingival fibroblasts Human Pulp Fibroblasts	1. Cytokine/growth factor secretion 2. Hydrolase activity	1. Human cytokine antibody detection kit 2. spectrophotometrically (*p*-nitrophenyl butyrate)	TEGDMA induced the secretion of the cytokine MCP-1 from U937 cells and also increased the hydrolase activity in the HGF. These results showed that TEGDMA induces enzymatic activity and cytokine/growth factor expression in a cell-specific manner.
Eckhardt *et al.* 2009 [122]	TEGDMA (0–2 mM)	Murine RAW264.7 macrophages	1. Cell survival 2. Cytokine release (TNF-a, IL-6, IL-10) 3. Expression of cell surface antigens (CD14, CD40, CD80, CD86, CD54, MHC class I, II)	1. Crystal violet staining 2. ELISA 3. FACs	TEGDMA resulted in inhibition of LPS-induced release of TNF-a, IL-6, and IL-10 by 90%. The expression of CD14 was inhibited by high TEGDMA concentrations. CD40, CD80, CD86 and MHC class I were also down-regulated. On the contrary, CD54 was increased about twofold by increasing TEGDMA concentrations. Thus, the ability of macrophages to induce an appropriate immune response is inhibited by TEGDMA.
Lee *et al.* 2009 [145]	HEMA (0–12 mM) TEGDMA (0–3 mM)	Murine RAW264.7 macrophages	1. Cell viability 2. COX-2 and iNOS gene expression 3. COX-2 protein expression	1. WST-8 assay 2. RT-PCR 3. WB	It was found that COX-2 expression was stimulated by TEGDMA and HEMA. PGE2 was produced by TEGDMA but not by HEMA in the murine cell line. These findings suggest that TEGDMA and HEMA can be a critical factor of inflammation related to resin-based dental biomaterials.
Imazato *et al.* 2009 [160]	TEGDMA (100–10 μg/mL), MMA (10–1 μg/mL) HEMA (400–50 μg/mL)	Osteoblast-like MC3T3-E1 cells	1. Cytotoxicity 2. Cell morphology 3. ALP Activity 4 Differentiation 5. Mineralized Tissue	1. MTT 2. SEM 3. Spectrophotometrically 4. RT-PCR 5. Alisarin Red staining	TEGDMA and MMA did not affect the growth of MC3T3-E1 and exhibited little harmful effects on their differentiation and mineralization. On the contrary, HEMA inhibited proliferation, ALP activities, the expression of osteocalcin, and mineralized tissue formation at 200 μg/mL or more.
Chang *et al.* 2009 [155]	BisGMA (0.025–0.2 mM)	Human Pulp Fibroblasts (HPFs)	1. Cytotoxicity with/without aspirin, catalase, and U0126 2. PGE2 production 3. COX-2 mRNA & protein expression and ERK1/2 phosphorylation 4. ROS production	1. MTT 2. ELISA 3. RT-PCR, WB 4. FACs (DCFH-DA)	BisGMA (>0.075 mM) induced cytotoxicity to HPFs. BisGMA (0.1 mM) also stimulated ERK phosphorylation, PGE2 production, COX-2 mRNA and protein expression, as well as ROS production. Catalase and U0126 (a MEK inhibitor) effectively prevented these phenomena Moreover, catalase can protect the pulp cells from BisGMA cytotoxicity, whereas aspirin and U0126 lacked of this protective activity.

**Table 2.** Mechanisms of genotoxic effects of substances released by resin-based dental restorative materials.

**Study**	**Substances studied (concentration)**	**Cell line**	**Biological parameters assessed**	**Methods**	**Main conclusions**
Schweikl *et al.* 1999 [138]	TEGDMA	V79 Chinese hamster lung fibroblasts	A. Genotoxicity B. hprt expression	1. Micronucleus test 2. PCR	TEGDMA induced dose-dependent increase of micronuclei and hprt deletions in a total of 24 cell clones.
Li *et al.* 1999 [139]	HEMA	Human Pulp Fibroblasts (HPF)	1.cytotoxicity 2. Cell cycle	1. MTT 2. FACs (PI)	HEMA induced dose dependent cytotoxicity and cell cycle arrest in G2 phase.
Schweikl *et al.* 2001 [104]	BisGMA (0–0.075 mM) UDMA (0–0.075 mM) HEMA (0–5 mM) TEGDMA (0–1 mM) GMA (0–0.2 mM) MMA (0–30 mM) BPA (0–0.2 mM)	V79 Chinese hamster lung fibroblasts	1. Cytotoxicity 2. Genotoxicity	1. Crystal Violet staining 2. Micronucelus test *in vitro* (in presence or absence of mix)	The cytotoxicity ranking was BisGMA > UDMA > BPA > GMA >>> TEGDMA >>> HEMA > MMA. A dose-related increase of micronuclei was observed by TEGDMA, HEMA and GMA. These effects were reduced by S9 mix.
Kostoryz *et al.* 2003 [158]	Bis-GMA, BFDGE & metabolites (0.001– 10 mM)	L-929 mouse fibroblasts MCF- 7 human breast cancer cells	1.cytotoxicity 2.mutagenesis 3.estrogenic effects	1. MTT 2. Ames test 3. Cell proliferation	Hydroxylized metabolites of Bis-GMA & BFDGE were less cytotoxic than initial monomers and presented no mutagenic or estrogenic effects.
Kleinsasser *et al.* 2004 [137]	UDMA, TEGDMA HEMA, BisGMA (10^−8^ 10^−7,^ 10^−6^ 10^−5,^ 10^−4^ 10^−3^ 10^−2^ and 2.5x10^−2^ M)	Human peripheral lymphocytes	1. Cytotoxicity 2. Genotoxicity	1. Trypan Blue 2. Single gel electrophoresis (Comet) assay	At higher concentrations, the monomers tested induced significant but mild enhancement of DNA migration in the Comet assay, as a possible sign for limited genotoxic effects.
Lee *et al.* 2006 [109]	HEMA (1–18 mM) TEGDMA (0.4–5 mM) GMA (0.08–0.8mM) in presence of NAC (10 mM)	V79 Chinese hamster lung fibroblasts RPC-C2 Rat clonal dental pulp cells	1. Cytotoxicity 2. Genotoxicity 3. Apoptosis	1. MTT 2. Micronucleus test & DNA gel electrophoresis 3. Flow cytometry (Annexin V-PI)	All monomers exhibited dose-dependent cytotoxic and genotoxic effects, with the following ranking GMA > TEGDMA > HEMA. These effects were significantly reduced in the presence of NAC.
Kleinsasser *et al.* 2006 [136]	UDMA, HEMA, TEGDMA (10^−7,^ 10^−5,^ 10^−3^, and 2.5x10^−2^ M)	Human samples of salivary glands and peripheral lymphocytes	1. Cytotoxicity 2. Genotoxicity	1. Trypan Blue 2. Single gel electrophoresis (Comet) assay	The monomers tested induced significant DNA migration in both cell types detected in the Comet assay even at non toxic concentrations. These genotoxic effects suggest a tumor initiating potency of the tested dental materials
Schweikl *et al.* 2007 [127]	HEMA (2–8 mM) TEGDMA (0.5–3 mM) in the presence of NAC (1, 5, 10 mM)	V79 Chinese hamster lung fibroblasts	1. Cell cycle 2. Genotoxicity	1. FACs (PI) 2. Micronucleus test *in vitro*	V79 cells were protected from genotoxicity and disruption of the cell cycle by TEGDMA and HEMA in the presence of high NAC concentrations (5, 10 mM).
Li *et al.* 2007 [165]	CQ ± DMT ± VL irradiation	Chinese hamster Ovary (CHO) cells	1. Genotoxicity 2. Cell cycle	1. Micronucleus tests 2. CBPI =Cytokinesis Block Proliferation Index	CQ/DMT with or without VL irradiation caused significant prolongation of the cell cycle. In addition, VL irradiated CQ/DMT was found to exhibit significantly genotoxic and cytotoxic effects, compared with CQ/DMT alone. These effects were reduced by pre-treatment with antioxidants.
Eckhardt *et al.* 2009 [143]	TEGDMA (0–5 mM)	THP-1 monocytes	1. Cell viability 2. DNA damage 3. Cell cycle 4. Detection of pATM, phospho-p38 and phospho-ERK1/2	1. MTT 2. Detection of 8-oxoguanine (OxyDNA Assay) 3. FACs (PI) 4. FACs analysis (antibodies)	TEGDMA induced oxidative DNA damage followed by activation of ATM and various signal transduction pathways through MAP kinases which also regulate cell death and survival.

## Abbreviations:

ALP: Alkaline Phosphatase
Bis-GMA: 2,2-bis[4-(2-hydroxy-3-methacryloxypropoxy)phenyl]propane)
BP: Benzoyl Peroxide
BPA: Bisphenol A
BrdU: 5-bromo-2’-deoxyuridine
CCCP: carbonylcyanide *m*-chlorophenylhydrazone
CMF-DA: Chloromethylfluorescein diacetate
CQ: camphorquinone
DCFH-DA: 2’,7’-dichlorofluorescein diacetate
DMA: 2-dimethylaminoethyl methacrylate
DMABEE: 4-*N,N*-Dimethylaminobenzoic acid ethylester
DMT: *N,N*-dimethyl-*p*-toluidine
EMSA: Electromobility shift assay
FACs: Flow cytometry
GSH: Glutathione
HEMA: 2-hydroxyethylmethacrylate
HMBP: 2-hydroxy-4-methoxybenzophenone
LPS: lipo-polysaccharide
MBBr: monobromobimane
mBCl: monochlorobimane
MTT: 3-(4,5-dimethylthiazol-2-yl)-2,5-diphenyltetrazoliombromide
NAC: *N*-acetylcysteine
PI: propidium-iodide
PI3-Kinase: phosphatidylinositol 3 kinase
ROS: Reactive Oxygen Species
S9 mix: metabolically active microsomal fraction from mouse or rat liver
SDS-PAGE electrophoresis: Sodium Dodecyl Sulfate Polyacrylamide gel electrophoresis
SEM: Scanning Electron Microscopy
TBARS: Thiobarbituric acid reactive substances
TEGDMA: triethyleneglycoldimethacrylate
TUNEL assay: terminal deoxyribonucleotidyl transferase (TdT) uridine triphosphate
UTP: (UTP) nick-end labeling
UDMA: urethanedimethacrylate
WB: Western blotting
WST-8: [2-(2-methoxy-4-nitrophenyl)-3-(4-nitrophenyl)-5-(2,4-disulfophenyl)-2*H*-tetrazolium, monosodium salt]
